# Clinicopathological significance of platelet-derived growth factor (PDGF)-B and vascular endothelial growth factor-A expression, PDGF receptor-β phosphorylation, and microvessel density in gastric cancer

**DOI:** 10.1186/1471-2407-10-659

**Published:** 2010-11-30

**Authors:** Shioto Suzuki, Yoh Dobashi, Yayoi Hatakeyama, Ryosuke Tajiri, Takashi Fujimura, Carl H Heldin, Akishi Ooi

**Affiliations:** 1 Department of Molecular and Cellular Pathology, Kanazawa University Graduate School of Medical Science, Ishikawa, Japan; 2 Department of Pathology, Saitama Medical Center, Jichi Medical University, Saitama, Japan; 3 Gastroenterologic Surgery, Kanazawa University Graduate School of Medical Science, Ishikawa, Japan; 4 Ludwig Institute for Cancer Research, Uppsala University, Uppsala, Sweden

## Abstract

**Background:**

Angiogenesis is important in the growth and metastasis of various kinds of solid tumors, including gastric cancers. The angiogenic process is triggered by several key growth factors, including vascular endothelial growth factor (VEGF)-A and platelet-derived growth factor (PDGF)-B, that are secreted by tumors. Our aim was to define: i) the expression pattern of VEGF-A and PDGF-B in tumor cells and the activation of PDGF receptor (PDGFR)-β tyrosine kinase in stromal cells of human gastric adenocarcinomas; and ii) the relationship between VEGF-A and PDGF-B expression and microvessel density (MVD), to determine if there is a rationale for a new therapeutic strategy.

**Methods:**

A series of 109 gastric adenocarcinoma cases that had undergone surgical resection was examined immunohistochemically using antibodies against VEGF-A, PDGF-B, and CD34, followed by further examination of PDGFR-β phosphorylation by immunoblotting analysis.

**Results:**

MVD was higher in diffuse-type than intestinal-type cancers (p < 0.001). VEGF-A overexpression correlated to PDGF-B overexpression in both the intestinal-type (p < 0.005) and diffuse-type (p < 0.0001) groups, indicating that VEGF-A and PDGF-B are secreted simultaneously in the same tumor, and may thus play important roles together in angiogenesis. However, several differences between intestinal-type and diffuse-type cancers were observed. In the diffuse-type cancer group, higher MVD was related to the PDGF-B proportion (p < 0.05) and VEGF-A overexpression (p < 0.05), but not to PDGF-B overexpression or the VEGF-A proportion. On the other hand, in the intestinal-type cancer group, higher MVD was correlated to overexpression (p < 0.005), intensity (p < 0.05), and proportion (p < 0.05) of PDGF-B, but not of VEGF-A. In addition, phosphorylation of PDGFR-β was correlated with depth of cancer invasion at statistically significant level.

**Conclusions:**

Our results indicate that PDGF-B, which is involved in the maintenance of microvessels, plays a more important role in angiogenesis in intestinal-type gastric carcinomas than VEGF-A, which plays a key role mainly in the initiation of new blood vessel formation. In contrast, VEGF-A has a critical role for angiogenesis more in diffuse-type cancers, but less in those of intestinal type. Thus, a therapy targeting the PDGF-B signaling pathway could be effective for intestinal-type gastric carcinoma, whereas targeting VEGF-A or both VEGF-A and PDGF-B signaling pathways could be effective for diffuse-type gastric carcinomas.

## Background

Over at least the past five decades, the mortality associated with gastric cancer has decreased markedly in most areas of the world [1,2]. However, gastric cancer remains one of the most common human malignancies worldwide [3]. The overall prognosis of gastric cancers still remains unsatisfactory, although recent surgical and chemotherapeutic interventions prolong survival of patients in advanced stages [2]. Thus, improvement of gastric cancer therapy will depend on early detection and novel therapeutic approaches. One of the potentially useful approaches is to inhibit tumor angiogenesis. In an attempt to precisely evaluate angiogenesis and its inhibition, the degree of tumor angiogenesis has been estimated by microvessel density (MVD). MVD, measured by the hot spot method, is a valuable prognostic indicator for a wide range of tumor types [4-6]. Previous studies showed that the angiogenic process is triggered by several key growth factors that are secreted by the tumor [7,8]. Among them, vascular endothelial growth factor (VEGF)-A and platelet-derived growth factor (PDGF)-B are the most studied [7,9-13]. It has been demonstrated that these two growth factors participate in the angiogenic process, and that VEGF-A plays a key role mainly in the initiation of the formation of new blood vessels, whereas PDGF-B is involved in the maintenance of microvessels and recruitment of pericytes [7]. These observations prompted an interest in designing strategies to suppress the functions of VEGF-A and PDGF-B, with the ultimate goal of inhibiting angiogenesis and starving tumors. These strategies include inhibition of the binding of VEGF-A and PDGF-B to their respective receptors using antibodies against the growth factors. One of these, bevacizumab (Avastin), which targets VEGF-A, has recently been approved for clinical use in patients with metastatic colorectal cancer, as well as non-small cell lung cancer [14,15]. Another approach has involved the development of inhibitors of the tyrosine kinase activities of the PDGF-B and VEGF-A receptors, which suppress the downstream signal transduction pathways triggered by these growth factors [15,16]. Most of these agents mimic the structure of ATP, and some are potent antitumor agents that are presently in clinical trials. However, none has yet been approved for gastric cancers [17].

Previous reports focused on the role of VEGF-A in gastric carcinomas and demonstrated that positive immunohistochemical staining for VEGF-A correlates with lymph node metastasis, depth of invasion, and vascular invasion, suggesting that VEGF-A might be a useful biomarker of tumor aggressiveness [18-21]. However, other reports found no significant association between VEGF-A expression and disease progression or patient overall survival [22,23], or that VEGF-A expression was more common in tumors without serosal invasion [24]. Furthermore, several reports showed higher VEGF-A expression in intestinal-type than diffuse-type gastric adenocarcinoma [22,25], whereas another study reported that VEGF-A expression was not related to histological type of gastric cancers [26]. Thus, the role of VEGF-A in gastric carcinomas remains controversial.

A few reports have focused on the expression of PDGF isoforms or their receptors in gastric adenocarcinomas [27-29]. However, the role of the PDGF-B signal pathway in gastric carcinoma has not yet been explained.

Our aim was to define: i) the expression pattern of PDGF-B and VEGF-A in tumor cells and activation of PDGFR-β tyrosine kinases in stromal cells of human gastric adenocarcinoma; and ii) the relationship between VEGF-A and PDGF-B expression and MVD, to determine whether there is a rationale for a new therapeutic strategy.

## Methods

### Tissue samples

A total of 109 cases of gastric cancers obtained from consecutive surgeries performed at the Department of Surgery, Kanazawa University, between 2003 and 2009 were examined (Table 1). The patients included 69 male and 40 female patients, with a mean age of 65.3 years (range, 26-85 years). The condition of the patients was assessed according to the system for staging primary tumor/regional lymph nodes/distant metastasis (TNM) described in the AJCC Cancer Staging Manual [30]. Tumors penetrating serosa or involved adjacent structures were observed in 44 patients (40.4%). Seventy five patients (68.8%) had lymph node metastases. According to Lauren's criteria [31], the specimens were classified into 63 intestinal-type and 46 diffuse-type adenocarcinomas. Thirty eight patients underwent total gastrectomy and 71 patients underwent partial resection for their cancers. D2 or more extended lymph node dissection was conducted for 96 patients (88.0%). All cancers were resected (no residual tumor, R-category 0) for 72 patients (66.1%). None of the patients had received any preoperative treatments, including neoadjuvant therapy. This laboratory study was approved by the Institutional Review Board at the Kanazawa University, and written informed consent was obtained from all patients.

**Table 1:** Clinicopathological features

	No. of patients	%
Total number of patients	109	
Sex		
Male	69	63.3
Female	40	36.7
Age		
Mean (Range)	65.3 (26-85)	
Lauren classification		
Intestinal	63	57.8
Diffuse	46	42.2
T stage		
T1	7	6.4
T2	58	53.2
T3	35	32.1
T4	9	8.3
N stage		
N0	34	31.2
N1	55	50.5
N2	14	12.8
N3	6	5.5
Extent of surgical resection		
gastrectomy	38	34.9
partial resection	71	65.1
Extent of lymphadenectomy		
D0	4	3.7
D1	8	7.3
D2	91	83.5
D3	5	4.6
Residual tumor status (R-category)		
R0	72	66.1
R1	27	24.8
R2	10	9.2

### Immunohistochemical staining

The Universal Immuno-Enzyme Polymer method (Nichirei simple staining) was used. Tissue samples of the investigated patients were obtained from the Pathology Department of our university. The most invasive areas of the carcinoma were selected; formalin-fixed and paraffin-embedded blocks of those were cut 3-μm-thick and used for further immunostaining. Sections were stained with 0.02% diaminobenzidine (DAB) solution, followed by counterstaining with hematoxylin. Primary antibodies used were a mouse monoclonal immunoglobulin (Ig) G specific for PDGF-B (PGF007, monoclonal; Mochida; dilution 1:1000), CD34 (monoclonal, Dako, ready-to-use), α-smooth muscle actin (SMA) (clone 1A4, monoclonal, Dako, ready-to-use), HIF-1α (clone H1α 67, monoclonal, Novus Biologicals, dilution 1:50) and NG2 (monoclonal, Abcam, dilution 1:100), rabbit polyclonal IgG specific for VEGF (polyclonal, Lab Vision; 1:100) and rabbit monoclonal IgG specific for PDGFR-β (rabbit monoclonal; Cell Signaling Technology; 1:2000). Histofine Simple Stain Max PO (Multi) was used as a secondary antibody (Nichirei).

### Staining analysis

For VEGF-A and PDGF-B assessment, the staining intensity and the proportion of stained tumor cells were analyzed, since it has been a consensus that both variables should be quantitatively analyzed to evaluate the expression level of growth factors in correlation with angiogenesis within the tumor nodule [20,24]. Staining was considered immunoreactive when brown granules were identified in the cytoplasm or nucleus of tumor cells [24,27]. According to one of the established methods [20,24], staining intensity was scored as 0 (none), 1+ (weak), 2+ (moderate), or 3+ (strong). The proportion of positively stained tumor cells in lesions was scored as 0 (0%), 1 (1%-25%), 2 (26-50%), 3 (51-75%), or 4 (76%-100%). When the sum of the two scores was less than 4, the section was considered negative, whereas 4 or more was considered positive for overexpression of VEGF-A or PDGF-B; the average values for both were between 3 and 4 (3.7 for PDGF-B and 3.3 for VEGF-A).

To assess tumor angiogenesis, MVD was determined by immunohistochemical staining of CD34. The generally accepted criteria for determining a vessel profile [5,6] were used, including any stained endothelial cell or endothelial cell cluster that was separate from adjacent microvessels. Vessel lumens were not required for identifying a structure as a microvessel. Microvessels in necrotic or sclerotic areas within a tumor and immediately adjacent areas of unaffected gastric tissue were not considered in vessel evaluations. The amount of immunohistochemically highlighted microvessel profiles was subjectively categorized by MVD scores 1-3. Two observers performed the vascular scoring by scanning the tumor section at low magnifications, using ×4 and ×10 objective lens, thereby finding three separately located tumor areas, where the highest number of discrete microvessels was stained (hot-spots). Each hot-spot area was equivalent to a high power field with a ×25 objective lens and a field diameter of 0.50 mm. The vascular grading is both influenced by the number of vessel profiles in the initial scanning for hot-spots and by the area of the vessel profiles within the hot-spots in the successive grading process. Thus, given an area with high angiogenesis activity by many microvessels, the vessel profiles with a larger cross-sectional area or perimeter contribute more to a high vascular grade. Score 1 (low angiogenesis) was registered when the combined area of the three hotspots contained a low amount of endothelial-stained microvessel profiles. Score 1 was typically assigned to tumors without any actual hot-spots. Score 2 (intermediate angiogenesis) was assigned when the combined area of the three hot-spots contained a moderate amount of vessel profiles. Score 2 was typically assigned to tumors with one very vascular hot-spot or with two hot-spots with only a low amount of microvessels. Score 3 (high angiogenesis) was registered when the combined area of the three hot-spots had numerous vessel profiles with a large average area or perimeter of vessel profiles. The determination of angiogenesis was performed without knowledge of the prognostic outcome. About one minute was used for vascular grading per tumor.

The number and location of pericytes were determined by combined assessment of immunohistochemical results against PDGFR-β, αSMA and NG2.

According to a previous report [22], a positive value for HIF-1α was recorded when nuclear staining was observed in >1% of cancer cells, whereas cytoplasmic staining was not counted.

Assessment of the staining was scored independently by two investigators (S.S. and A.O.) without knowledge of the clinicopathological findings. The allocation of tumors and scoring of staining by the two investigators was similar. In cases of disagreement, slides were reevaluated and discussed until consensus was achieved.

### Immunoblotting analysis

Lysates were prepared from these fresh tissues as described [32], and immunoblotting analysis was performed. Equal amounts (30 μg of lysates) of protein were used for blotting with anti-PDGF-B (PDGF-BB, Abcam; 1:200) and anti-p-PDGFR-β (Tyr751, Cell Signaling Technology; 1:2000) antibodies. Blotting with anti-β-actin (Ambion; 1:5000) and anti-PDGFR-β (rabbit monoclonal; Cell Signaling Technology; 1:2000) antibodies was also performed as loading controls.

Expression levels were quantified by densitometric analysis with Chemi Imager 5500 (AlphaInotech). Levels of PDGF-B or p-PDGFR-β were standardized by β-actin or PDGFR, respectively, assigned an arbitrary level of "1.0"; the expression signal relative to these was indicated as the "expression value" for each protein. The "protein index" of PDGF-B or p-PDGFR-β was obtained by dividing the "expression value" in tumor tissue by that in non-neoplastic tissue. In this study, expression signal was interpreted as "overexpressed" (for PDGF-B expression) or "activated" (for p-PDGFR-β) i) when the "protein index" was higher than 1.5 or 1.0, respectively, or ii) when protein expression was barely detectable in the paired non-neoplastic tissue [32,33].

### Statistical analysis

StatView software (version 5.0; Abacus Concepts) was used for the data analysis. Clinicopathological variables, as well as expression of VEGF-A and PDGF-B and MVD, were analyzed. The correlations between VEGF-A and PDGF-B expressions, MVD, and the other variables were assessed with the χ^2 ^and Fisher's exact tests. The Spearman test was performed to evaluate rank data. Survival durations were calculated via the Kaplan-Meier method. The log-rank test was employed to compare cumulative survival in the patient groups. Statistical significance was defined as a probability value less than 0.05, in all tests.

Agreement among observers for the interpretation of IHC specimens was qualified by kappa (κ) statistics [34]. In accordance with the criteria of Landis and Koch [35], the κ-values were divided into several scales to evaluate the strength of agreement: κ < 0.00, poor; 0.00<κ < 0.20, slight; 0.21<κ < 0.40, fair; 0.41<κ < 0.60, moderate; 0.61<κ < 0.80, substantial; 0.81<κ < 1.00, nearly perfect.

## Results

### Immunohistochemical analyses

#### MVD

**Figure 1:**
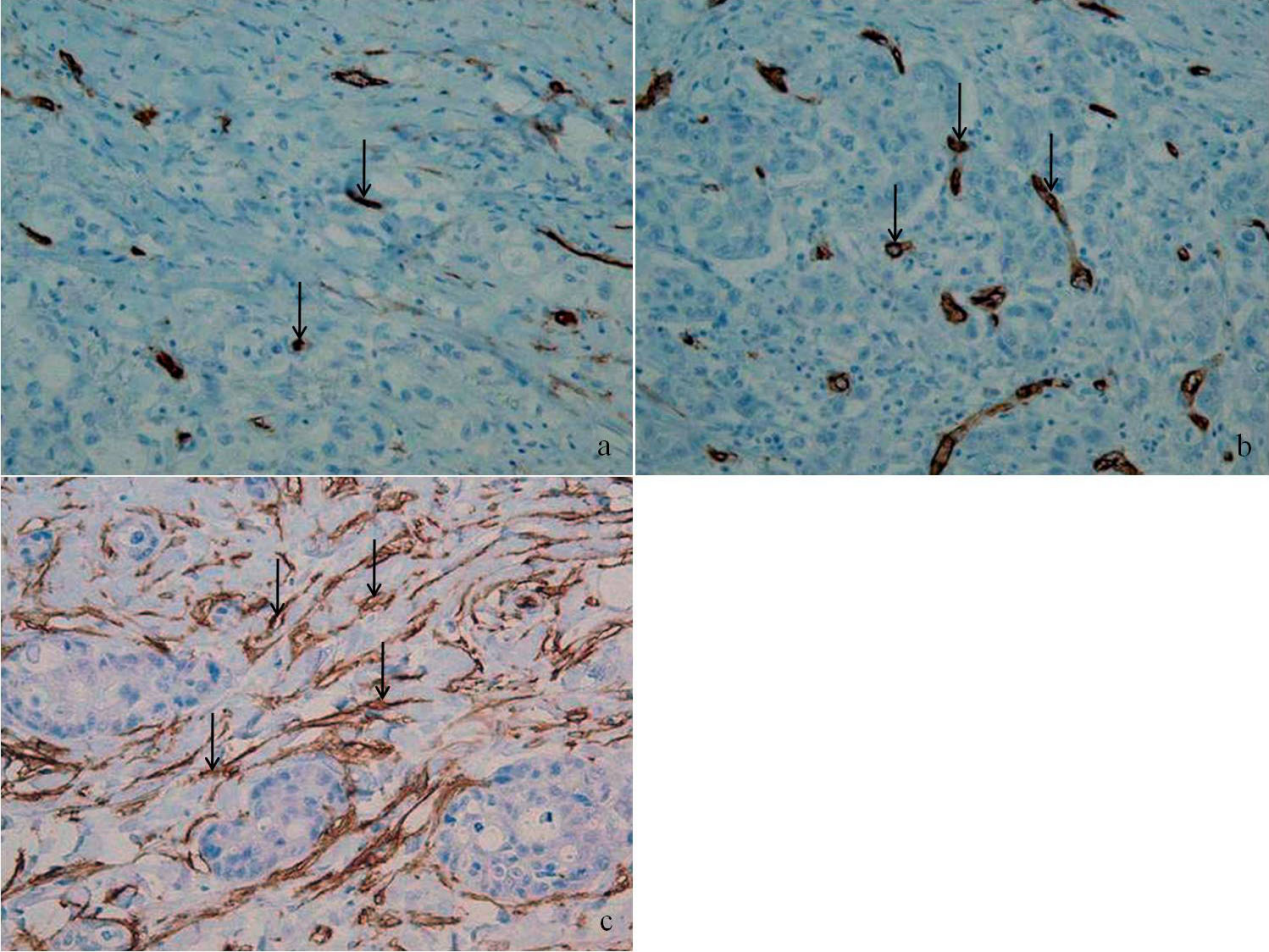
**Microvessel density staining for CD34 in intestinal type gastric cancer**. A hot-spot with low angiogenesis (a) contains fewer positive staining (arrow) than those of hot spot with intermediate (b) or high (c) angiogenesis. The vascular grade of the tumor would be intermediate or grade 2, if the three hot-spots were from the same tumor.

Of the 63 patients with intestinal-type cancers, 15 (23.8%) had an MVD score 1 (Figure 1a), 33 (52.3%) score 2 (Figure 1b), and 15 (23.8%) score 3 (Figure 1c), whereas of the 46 patients with diffuse-type cancers, 3 (6.5%) had score 1, 19 (41.3%) score 2, and 24 (52.1%) score 3. Overall interobserver agreement was nearly perfect (κ = 0.91; 95% confidence interval, 0.87-0.97). A higher MVD score was observed in the diffuse-type (mean 2.46) than intestinal-type (mean 2.00) cancers (p < 0.001).

### Expression of PDGF-B and VEGF-A

**Figure 2:**
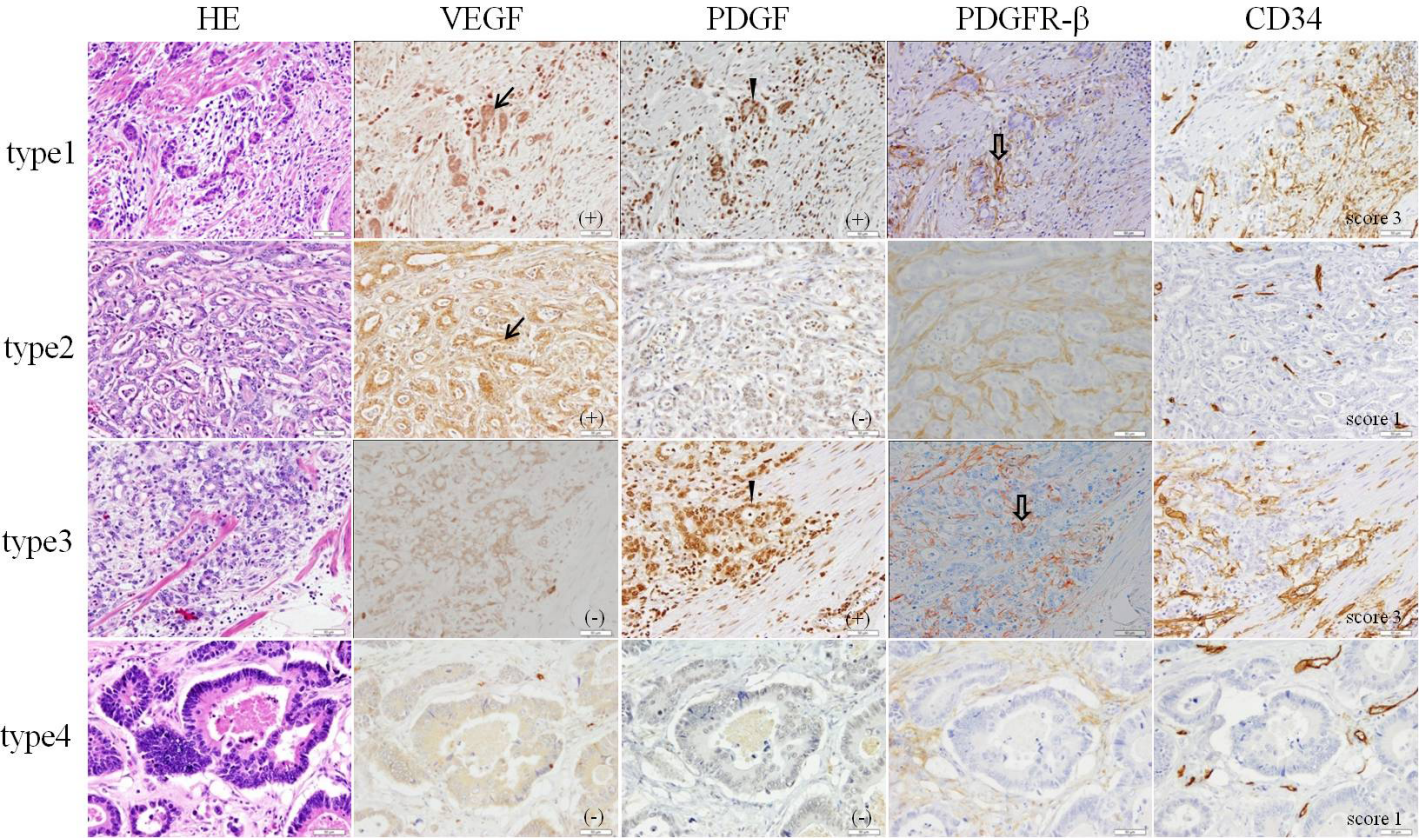
**Immunohistochemical staining of VEGF-A, PDGF-B, PDGFR-β and CD34 with hematoxylin and eosin staining (HE) in representative tissue sections obtained from human gastric carcinoma**. According to the presence/absence in overexpression of VEGF and PDGF, as measured by a summation score of staining intensity and proportion of positive staining cells for VEGF-A and PDGF-B, cases were classified to 4 types: Type1, VEGF-A+/PDGF-B+; Type2, VEGF-A+PDGF-B-; Type3, VEGF-A-/PDGF-B+; Type4, VEGF-A-/PDGF-B-. Immunoreactivity for VEGF-A is detected predominantly in the cytoplasm of the carcinoma cells (arrow), whereas PDGF-B reactivity is strong in the nuclei and weak in the cytoplasm (arrow head). Staining for PDGFR-β was seen in many pericytes (outline arrow) of PDGF-B overexpressing carcinomas, implying that PDGF produced by cancer cells caused increased pericyte coverage around vessels.

Immunoreactivity for VEGF-A was detected predominantly in the cytoplasm of the carcinoma cells (Figure 2). Immunoreactivity for PDGF-B was present diffusely or focally in the cytoplasm and nucleus of malignant cells, of some inflammatory cells, and of cells in the surrounding stroma (Figure 2), but not in normal epithelial cells. According to the presence/absence in overexpression of VEGF and PDGF, as measured by a summation score of staining intensity and proportion of positive staining cells, cases were classified to 4 types: Type 1, with overexpression of both VEGF-A and PDGF-B (36 cases); Type 2, with overexpression of VEGF-A but not PDGF-B (9 cases); Type 3, with overexpression of PDGF-B but not VEGF-B (19 cases); Type 4, without VEGF-A or PDGF-B overexpression (45 cases). Immunohistochemical analyses of representative cases in type1-4 are shown in Figure 2.

### Relationship between overexpression of PDGF-B and VEGF-A

In intestinal-type cancers, overexpression of PDGF-B or VEGF-A was observed in 50% (31 cases) or 41% (26 cases) of the 63 cases, respectively (Table 2). Overall interobserver agreement was nearly perfect (κ = 0.89; 95% confidence interval, 0.85-0.96). Overexpression of PDGF-B was more common in cases with overexpression of VEGF-A than in those without VEGF-A overexpression (p < 0.005, Table 2). In diffuse-type cancers, overexpression of PDGF-B or VEGF-A was observed in 52% (24 cases) or 41% (19 cases) of the 46 cases, respectively (Table 2). Overexpression of PDGF-B correlated with that of VEGF-A (p < 0.0001, Table 2).

**Table 2:** Relationships between PDGF overexpression or VEGF overexpression

	VEGF over expression		
	
< intestinal type >	(+)	(-)	
PDGF overexpression			
(+)	19	12	p < 0.005
(-)	7	25	

total	26	37	
			
< diffuse type >			

PDGF overexpression			
(+)	17	7	p < 0.0001
(-)	2	20	

total	19	27	

### Impact of VEGF-A and PDGF-B overexpression on angiogenesis

Analysis of the data according to histological type of carcinomas (intestinal vs. diffuse) showed some significant correlations that were not present when considering the global patient population.

For intestinal-type cancers, the following correlations were found. A higher MVD score correlated with proportion (p < 0.05), intensity (p < 0.05), and overexpression (p < 0.005, Table 3) of PDGF-B. However, the MVD score did not correlate with proportion (p = 0.67), intensity (p = 0.45), or overexpression (p = 0.72, Table 3) of VEGF-A. For diffuse-type cancers, a higher MVD score was correlated with proportion (p < 0.05) of PDGF-B, but not with intensity (p = 0.67) or overexpression (p = 0.15, Table 3) of PDGF-B staining. However, with regard to VEGF-A, the MVD score was correlated with overexpression (p < 0.05, Table 3), but not with proportion (p = 0.066) or staining intensity (p = 0.72).

**Table 3:** Relationships between MVD and overexpression of PDGF-B or VEGF-A

	VEGF overexpression		PDGF overexpression	
	
	(+)	(-)	(+)	(-)
<intestinal type>				
MVD	*p = 0.72		**p < 0.005	
1	7	8	4	11
2	11	22	15	18
3	8	7	12	3

<diffuse type>				
MVD	*p < 0.05		**p = 0.15	
1	0	3	0	3
2	6	13	10	9
3	13	11	14	10
total	19	27	24	22

*p, VEGF-A(+) group vs VEGF-A(-) group

**p, PDGF-B(+) group vs. PDGF-B(-) group

### Statistical analysis

In diffuse-type cancers, lymph node metastasis was correlated with a higher MVD score (p < 0.05), whereas the depth of invasion was not correlated with other factors. In intestinal-type cancers, prognostic factors, including lymph node metastasis and depth of invasion, did not correlate with PDGF-B and VEGF-A overexpression or the MVD score.

### Location of pericytes

To determine the number and location of pericytes, immunohistochemistry (IHC) was done with specimens using antibodies for αSMA, PDGFR-β and NG2. Positive stainings for PDGFR-β and αSMA were seen predominantly in the membrane and cytoplasm of the stromal cells, including pericytes, but not in carcinoma cells in similar patterns, although positive staining for NG2 was observed in only a few stromal cells. In detail, PDGFR-β staining was seen more selectively in cells around vessels, whereas αSMA staining was observed also in many other stromal cells. These findings showed that PDGFR-β had high specificity for recognizing pericytes. Furthermore, staining for PDGFR-β was seen in many pericytes in PDGF-B overexpressing carcinomas, implying that PDGF-B produced by cancer cells caused increased pericyte coverage around vessels, whereas faint staining was also seen in pericytes of carcinomas without PDGF-B overexpression (Figure 2).

### Expression of HIF-1α

Cytoplasmic staining for HIF-1α was observed in many cancer cells, whereas nuclear staining was observed in only a part of them. The latter was located in both the center and the periphery of cancers and the location was not clearly related with staining for VEGF-A or PDGF-B.

### Prognostic significance of VEGF-A and PDGF-B overexpression and MVD

The median follow-up duration was 31 months (range, 1-85.4 months) after operation. The hospital mortality and postoperative morbidity were 0% and 4.5%, respectively. Recurrence of carcinomas were observed in 31 cases. No significant association was observed between survival and VEGF-A (p = 0.50) or PDGF-B (p = 0.91) overexpression or MVD (p = 0.73).

### Immunoblotting analysis

**Figure 3:**
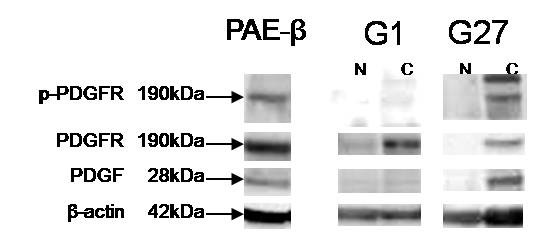
**Protein levels of PDGF-B, PDGFR-β, and p-PDGFR-β evaluated by immunoblotting analysis in representative two cases (G1, PDGF-B overexpression -/p-PDGFR-β-; G27, PDGF-B overexpression +/p-PDGFR-β +) of tumor and paired normal tissue**. Porcine aortic endothelial cells expressing PDGFR-β (PAE-β), was stimulated by PDGF-B and used as positive control for phosphorylation of PDGFR-β. N, normal tissue; C, cancer tissue.

To evaluate the relative levels of expression of PDGF-B or phosphorylation of PDGFR-β in tissue samples, and to evaluate their correlation with immunohistochemical results, immunoblotting was performed. The results from the 35 cases of fresh tissue specimens are presented in Table 4 and two representative tissue specimens are shown in Figure 3.

**Table 4:** Relationships between phosphorylation of PDGFR-β and PDGF-B overexpression

WB PDGFR-β phosphorylation	WB PDGF-B overexpression	IHC PDGF-B overexpression	No. of cases
+	+	+	5
+	+	-	4
+	-	+	3
+	-	-	1
-	+	+	11
-	+	-	8
-	-	+	1
-	-	-	2

Abbreviations: IHC, immunohistochemistry; WB, western blot.

PDGF-B was detectable as a 28-kD protein in all samples obtained from both tumor and normal tissues, upon analysis by SDS-gel electrophoresis under non-reducing conditions followed by immunoblotting. Of the 35 cases, 28 (80%) showed PDGF-B overexpression (more than 1.5 times of those of normal tissue). In 12 of these 28 cases, PDGF-B overexpression was detected by immunoblotting, but not by IHC, while in 4 cases, PDGF-B overexpression was detected by IHC, but not by immunoblotting.

PDGFR was detectable as a 190-kD protein in 17 samples obtained from both tumor and normal tissues. Activation of PDGFR-β in tumor, relative to normal tissue, was noted in 13 (37%) of the 35 cases. Among the 13 cases with activation of PDGFR, PDGF-B overexpression was detected by IHC or immunoblotting in 9 or 8 cases, respectively. No significant correlations were seen between activation of PDGFR-β and overexpression of PDGF-B detected by IHC or immunoblotting (p = 0.70 and p = 0.23, respectively).

### Relationship between activation of PDGFR-β and angiogenesis

There was no clear relationship between activation of PDGFR-β and angiogenesis in tumors (p = 0.55). Of the 13 tumors with PDGFR-β activation, 12 (92%) had a MVD score of 2 or 3, whereas in the 22 tumors without PDGFR activation, 18 (82%) had a MVD score of 2 or 3.

### Relationship between activation of PDGFR-β and depth of invasion

Among 20 cases with PDGF-B overexpression, detected by IHC, all 8 cases with activation of PDGFR-β, detected by immunoblotting, penetrated into the subserosal layer, whereas only 6 of 12 cases without activation of PDGFR-β invaded into the subserosal layer (p < 0.05). Thus, phosphorylation of PDGFR-β was correlated with depth of cancer invasion at statistically significant level.

## Discussion

There has been much literature describing the overexpression of VEGF-A in gastric cancer, with frequencies ranging from 36% to 76% [18-22,26,27,36], consistent with the 43.1% found in the present study.

Using IHC, previous studies showed that VEGF-A expression was seen more frequently in intestinal-type than in diffuse-type cancers [18,22,25]. In addition, using an enzyme immunoassay in gastric cancers and surrounding non-cancerous mucosa, another study showed that VEGF-A expression was significantly higher in intestinal-type than diffuse-type gastric cancer [37]. However, other studies reported that VEGF-A expression was not related to histological type of gastric cancer [24,26]. In the present study, no difference between the intestinal-type and diffuse-type cases was seen with regard to the frequency of VEGF-A overexpression (41.3% and 41.3%, respectively; p = 0.997) (Table 2).

A significant correlation between MVD score and VEGF-A expression has been reported in certain previous studies [18,20,27], but not in others [37]. The result of the present study is consistent with the latter (p = 0.14). However, analysis of the data according to histological type of carcinoma (intestinal vs. diffuse), VEGF-A overexpression, as measured by a summation score of staining intensity and proportion of positive staining cells for VEGF-A, was related with higher MVD in diffuse-type gastric cancers (p < 0.05), but not in intestinal-type cancers (p = 0.72) (Table 3). This result suggests that the ratio of diffuse-type cases relative to total cases may have influenced the relationship between VEGF-A expression and MVD score in the statistical analysis as in previous studies if all samples were analyzed as a whole. In the present study, diffuse-type cases represented 35% of male cases and 55% of female cases, which are close to the natural incidence [38].

In a previous study, it was demonstrated that the positive immunostaining rates of VEGF-A correlated with lymph node metastasis, depth of invasion, and vascular invasion, suggesting that this protein might be a useful biomarker of tumor aggressiveness [20]. However, other studies showed that high VEGF-A and MVD were more common in tumors without serosal invasion [24], and no significant correlation between VEGF-A expression and MVD score, patient survival, and clinicopathological factors was found [22,37]. Consistent with the latter studies, in the present study, VEGF-A overexpression was not correlated with lymph node metastases and depth of invasion and no association was found between patient survival and VEGF-A expression or MVD. However, phosphorylation of PDGFR-β was significantly correlated with depth of cancer invasion.

A few reports have focused on the expression of PDGF isoforms or their receptors in gastric adenocarcinomas [27-29] and thus, the role of the PDGF-B signal pathway in gastric carcinoma has not yet been explained. In the present study, IHC analysis showed that PDGFR-β staining had high specificity for recognizing pericytes, compared to NG2 and αSMA. This result was consistent with the result in one recent study showing that PDGFR-β expression was restricted to stromal pericytes [28]. Moreover, in the cases with carcinoma overexpressing PDGF-B, more intense PDGFR-β staining was seen in many pericytes than in those without PDGF-B overexpression. This result may suggest that PDGF-B, produced by cancer cells, cause increased pericyte coverage around vessels, and upregulation of PDGFR-β expression.

On the other hand, PDGF-B staining was not restricted in cancer cells and also in some inflammatory and stromal cells. In some cases, there was a discrepancy of the results obtained by immunoblotting compared to IHC (Table 4). One of the reasons for this may be that immunoblotting provides data of an average of expression levels of PDGF-B produced not only by cancer cells but also by inflammatory and stromal cells, and not a focal expression in cancer cells as evaluated with IHC.

Overall, our thorough study of the correlations of PDGF-B and VEGF-A with angiogenesis revealed several new findings. First, PDGF-B overexpression was seen in 50% of all cases and correlated with a higher MVD score. These results are almost consistent with those reported previously, showing PDGF-B expression in 41% of cases and its correlation with a higher MVD score [27]. Second, PDGF-B overexpression correlated with VEGF-A overexpression in intestinal-type and diffuse-type cancers (Table 2). These results suggest that PDGF-B and VEGF-A were secreted simultaneously in the same tumor and cooperated in the stimulation of angiogenesis. Finally, as novel findings, several differences between intestinal-type and diffuse-type cancers were observed. In the diffuse-type group, the MVD score was related with PDGF-B proportion and VEGF-A overexpression, but not with PDGF-B overexpression (Table 3) or VEGF-A proportion. On the other hand, in intestinal-type cancers, MVD score correlated with overexpression (Table 3), intensity, and proportion of PDGF-B, whereas no correlation was observed between a higher MVD score and overexpression, intensity, and proportion of VEGF-A.

Collectively, these results suggest that: i) VEGF-A is an important factor for angiogenesis, critically involved more in diffuse-type, but less in intestinal-type cancers; and ii) that PDGF-B plays an important role not only in intestinal-type but also diffuse-type cancers. Therefore, therapies targeting the PDGF-B signaling pathway could be effective for intestinal-type cancers, whereas therapies targeting VEGF-A or both VEGF-A and PDGF-B signaling pathways could be effective for diffuse-type gastric cancers. Recently, many inhibitors have been developed against PDGF-B, VEGF-A, and their cognate receptors. An inhibitor targeting VEGFRs in endothelial cells (SU5416) has been demonstrated to be effective against early-stage angiogenic lesions [16]. In contrast, a kinase inhibitor selective for PDGFRs (SU6668) was shown to block growth of end-stage tumors, eliciting detachment of pericytes and disruption of tumor vascularity [16]. Another study showed that treatment with the selective PDGF receptor kinase inhibitor, STI571 (imatinib), decreased interstitial hypertension and increased drug uptake and therapeutic effectiveness of cancer chemotherapy [39]. Thus, neoadjuvant therapy using these drugs may decrease tumor size, reduce the stage or extent of tumor before attempting surgical control, or improve the results of surgery, although no association was found between survival and overexpression of VEGF-A or PDGF-B in the current study. Furthermore, the result may help to stratify patients with diffuse or intestinal types of gastric carcinomas, before treatment or after the operation at the time of relapse, for treatment with kinase inhibitors, targeting PDGF-B or VEGF-A receptors. This kind of tailored preoperative regimens may enable a limited surgical intervention, such as endoscopic submucosal dissection or partial gastrectomy, and avoid needlessly treating the patients by total gastrectomy.

## Conclusions

PDGF-B plays a more important role in angiogenesis in intestinal-type gastric carcinomas than VEGF-A. In contrast, VEGF-A has a more critical role for angiogenesis in diffuse-type cancers. Thus, a therapy targeting the PDGF-B signaling pathway could be effective for intestinal-type gastric carcinoma, whereas targeting VEGF-A or both VEGF-A and PDGF-B signaling pathways could be effective for diffuse-type gastric carcinomas.

## Abbreviation

VEGF: vascular endothelial growth factor; PDGF: platelet-derived growth factor; MVD: microvessel density; PDGFR: platelet-derived growth factor receptor; IHC: immunohistochemistry.

## Competing interests

The authors declare that they have no competing interests.

## Authors' contributions

SS participated in the study design, carried out most of the experiments, performed all of the histological evaluation, including statistical analysis, and drafted the manuscript. YD has conceived of the study, participated in study design and coordination, and drafting of the manuscript. YH, RT and TF have made substantial contributions to acquisition of data, or analysis and interpretation of data and have been involved in revising the manuscript critically for important intellectual content. CHH was involved in study design and drafting of the manuscript. AO participated in the study design, performed the histological evaluation and drafted the manuscript. All authors read and approved the final manuscript.

## Pre-publication history

The pre-publication history for this paper can be accessed here:

http://www.biomedcentral.com/1471-2407/10/659/prepub

## References

[B1] Jemal A Siegel R Ward E Hao Y Xu J Thun MJ Cancer statistics, 2009 CA Cancer J Clin 2009 59 225 249 10.3322/caac.20006 19474385

[B2] Alberts SR Cervantes A van de Velde CJ Gastric cancer: epidemiology, pathology and treatment Ann Oncol 2003 14 Suppl 2 ii31 36 12810455 10.1093/annonc/mdg726

[B3] Parkin DM Bray F Ferlay J Pisani P Global cancer statistics, 2002 CA Cancer J Clin 2005 55 74 108 10.3322/canjclin.55.2.74 15761078

[B4] Hlatky L Hahnfeldt P Folkman J Clinical application of antiangiogenic therapy: microvessel density, what it does and doesn't tell us J Natl Cancer Inst 2002 94 883 893 12072542 10.1093/jnci/94.12.883

[B5] Hansen S Grabau DA Sorensen FB Bak M Vach W Rose C Vascular grading of angiogenesis: prognostic significance in breast cancer Br J Cancer 2000 82 339 347 10.1054/bjoc.1999.0924 10646886 PMC2363269

[B6] Weidner N Folkman J Pozza F Bevilacqua P Allred EN Moore DH Meli S Gasparini G Tumor angiogenesis: a new significant and independent prognostic indicator in early-stage breast carcinoma J Natl Cancer Inst 1992 84 1875 1887 10.1093/jnci/84.24.1875 1281237

[B7] Carmeliet P Angiogenesis in health and disease Nat Med 2003 9 653 660 10.1038/nm0603-653 12778163

[B8] Yancopoulos GD Davis S Gale NW Rudge JS Wiegand SJ Holash J Vascular-specific growth factors and blood vessel formation Nature 2000 407 242 248 10.1038/35025215 11001067

[B9] Nissen LJ Cao R Hedlund EM Wang Z Zhao X Wetterskog D Funa K Brakenhielm E Cao Y Angiogenic factors FGF2 and PDGF-BB synergistically promote murine tumor neovascularization and metastasis J Clin Invest 2007 117 2766 2777 10.1172/JCI32479 17909625 PMC1994630

[B10] Cao R Bjorndahl MA Religa P Clasper S Garvin S Galter D Meister B Ikomi F Tritsaris K Dissing S PDGF-BB induces intratumoral lymphangiogenesis and promotes lymphatic metastasis Cancer Cell 2004 6 333 345 10.1016/j.ccr.2004.08.034 15488757

[B11] Cao R Brakenhielm E Pawliuk R Wariaro D Post MJ Wahlberg E Leboulch P Cao Y Angiogenic synergism, vascular stability and improvement of hind-limb ischemia by a combination of PDGF-BB and FGF-2 Nat Med 2003 9 604 613 10.1038/nm848 12669032

[B12] Cao Y Cao R Hedlund EM R Regulation of tumor angiogenesis and metastasis by FGF and PDGF signaling pathways J Mol Med 2008 86 785 789 10.1007/s00109-008-0337-z 18392794

[B13] Cao Y Opinion: emerging mechanisms of tumour lymphangiogenesis and lymphatic metastasis Nat Rev Cancer 2005 5 735 743 10.1038/nrc1693 16079909

[B14] Cabebe E Wakelee H Role of anti-angiogenesis agents in treating NSCLC: focus on bevacizumab and VEGFR tyrosine kinase inhibitors Curr Treat Options Oncol 2007 8 15 27 10.1007/s11864-007-0022-4 17634832

[B15] Becker JC Muller-Tidow C Serve H Domschke W Pohle T Role of receptor tyrosine kinases in gastric cancer: new targets for a selective therapy World J Gastroenterol 2006 12 3297 3305 16733844 10.3748/wjg.v12.i21.3297 PMC4087885

[B16] Bergers G Song S Meyer-Morse N Bergsland E Hanahan D Benefits of targeting both pericytes and endothelial cells in the tumor vasculature with kinase inhibitors J Clin Invest 2003 111 1287 1295 12727920 10.1172/JCI17929 PMC154450

[B17] Valverde CM Macarulla T Casado E Ramos FJ Martinelli E Tabernero J Novel targets in gastric and esophageal cancer Crit Rev Oncol Hematol 2006 59 128 138 10.1016/j.critrevonc.2006.02.001 16829119

[B18] Zhao HC Qin R Chen XX Sheng X Wu JF Wang DB Chen GH Microvessel density is a prognostic marker of human gastric cancer World J Gastroenterol 2006 12 7598 7603 17171787 10.3748/wjg.v12.i47.7598 PMC4088040

[B19] Saito H Osaki T Murakami D Sakamoto T Kanaji S Ohro S Tatebe S Tsujitani S Ikeguchi M Prediction of sites of recurrence in gastric carcinoma using immunohistochemical parameters J Surg Oncol 2007 95 123 128 10.1002/jso.20612 17262742

[B20] Kolev Y Uetake H Iida S Ishikawa T Kawano T Sugihara K Prognostic significance of VEGF expression in correlation with COX-2, microvessel density, and clinicopathological characteristics in human gastric carcinoma Ann Surg Oncol 2007 14 2738 2747 10.1245/s10434-007-9484-7 17687613

[B21] Lieto E Ferraraccio F Orditura M Castellano P Mura AL Pinto M Zamboli A De Vita F Galizia G Expression of vascular endothelial growth factor (VEGF) and epidermal growth factor receptor (EGFR) is an independent prognostic indicator of worse outcome in gastric cancer patients Ann Surg Oncol 2008 15 69 79 10.1245/s10434-007-9596-0 17896140

[B22] Oh SY Kwon HC Kim SH Jang JS Kim MC Kim KH Han JY Kim CO Kim SJ Jeong JS Kim HJ Clinicopathologic significance of HIF-1alpha, p53, and VEGF expression and preoperative serum VEGF level in gastric cancer BMC Cancer 2008 8 123 10.1186/1471-2407-8-123 18452596 PMC2397429

[B23] Bennett C Paterson IM Corbishley CM Luqmani YA Expression of growth factor and epidermal growth factor receptor encoded transcripts in human gastric tissues Cancer Res 1989 49 2104 2111 2702651

[B24] Cabuk D Basaran G Celikel C Dane F Yumuk PF Iyikesici MS Ekenel M Turhal NS Vascular endothelial growth factor, hypoxia-inducible factor 1 alpha and CD34 expressions in early-stage gastric tumors: relationship with pathological factors and prognostic impact on survival Oncology 2007 72 111 117 10.1159/000111118 18025805

[B25] Mattioli E Vogiatzi P Sun A Abbadessa G Angeloni G D'Ugo D Trani D Gaughan JP Vecchio FM Cevenini G Immunohistochemical analysis of pRb2/p130, VEGF, EZH2, p53, p16(INK4A), p27(KIP1), p21(WAF1), Ki-67 expression patterns in gastric cancer J Cell Physiol 2007 210 183 191 10.1002/jcp.20833 16998811

[B26] Choi JH Ahn MJ Park CK Han HX Kwon SJ Lee YY Kim IS Phospho-Stat3 expression and correlation with VEGF, p53, and Bcl-2 in gastric carcinoma using tissue microarray Apmis 2006 114 619 625 10.1111/j.1600-0463.2006.apm_401.x 16948814

[B27] Tatsuguchi A Matsui K Shinji Y Gudis K Tsukui T Kishida T Fukuda Y Sugisaki Y Tokunaga A Tajiri T Sakamoto C Cyclooxygenase-2 expression correlates with angiogenesis and apoptosis in gastric cancer tissue Hum Pathol 2004 35 488 495 10.1016/j.humpath.2003.10.025 15116331

[B28] Drescher D Moehler M Gockel I Frerichs K Muller A Dunschede F Borschitz T Biesterfeld S Holtmann M Wehler T Coexpression of receptor-tyrosine-kinases in gastric adenocarcinoma--a rationale for a molecular targeting strategy? World J Gastroenterol 2007 13 3605 3609 17659711 10.3748/wjg.v13.i26.3605 PMC4146800

[B29] Chung CK Antoniades HN Expression of c-sis/platelet-derived growth factor B, insulin-like growth factor I, and transforming growth factor alpha messenger RNAs and their respective receptor messenger RNAs in primary human gastric carcinomas: in vivo studies with in situ hybridization and immunocytochemistry Cancer Res 1992 52 3453 3459 1317752

[B30] Greene FLPD Fleming ID eds AJCC Cancer Staging Manual 2002 6 Ner York: Springer-Verlag

[B31] Lauren P The Two Histological Main Types Of Gastric Carcinoma: Diffuse And So-Called Intestinal-Type Carcinoma. An Attempt At A Histo-Clinical Classification Acta Pathol Microbiol Scand 1965 64 31 49 14320675 10.1111/apm.1965.64.1.31

[B32] Suzuki S Igarashi S Hanawa M Matsubara H Ooi A Dobashi Y Diversity of epidermal growth factor receptor-mediated activation of downstream molecules in human lung carcinomas Mod Pathol 2006 19 986 998 10.1038/modpathol.3800619 16648865

[B33] Hoshino R Chatani Y Yamori T Tsuruo T Oka H Yoshida O Shimada Y Ari-i S Wada H Fujimoto J Kohno M Constitutive activation of the 41-/43-kDa mitogen-activated protein kinase signaling pathway in human tumors Oncogene 1999 18 813 822 10.1038/sj.onc.1202367 9989833

[B34] Thomson TA Hayes MM Spinelli JJ Hilland E Sawrenko C Phillips D Dupuis B Parker RL HER-2/neu in breast cancer: interobserver variability and performance of immunohistochemistry with 4 antibodies compared with fluorescent in situ hybridization Mod Pathol 2001 14 1079 1086 10.1038/modpathol.3880440 11706067

[B35] Landis JR Koch GG The measurement of observer agreement for categorical data Biometrics 1977 33 159 174 10.2307/2529310 843571

[B36] Nikiteas NI Tzanakis N Theodoropoulos G Atsaves V Christoni Z Karakitsos P Lazaris AC Papachristodoulou A Klonaris C Gazouli M Vascular endothelial growth factor and endoglin (CD-105) in gastric cancer Gastric Cancer 2007 10 12 17 10.1007/s10120-006-0401-8 17334712

[B37] Chen CN Hsieh FJ Cheng YM Cheng WF Su YN Chang KJ Lee PH The significance of placenta growth factor in angiogenesis and clinical outcome of human gastric cancer Cancer Lett 2004 213 73 82 10.1016/j.canlet.2004.05.020 15312686

[B38] Kaneko S Yoshimura T Time trend analysis of gastric cancer incidence in Japan by histological types, 1975-1989 Br J Cancer 2001 84 400 405 10.1054/bjoc.2000.1602 11161407 PMC2363747

[B39] Pietras K Ostman A Sjoquist M Buchdunger E Reed RK Heldin CH Rubin K Inhibition of platelet-derived growth factor receptors reduces interstitial hypertension and increases transcapillary transport in tumors Cancer Res 2001 61 2929 2934 11306470

